# Malignant lymphoma and *Schistosoma japonica* infection

**DOI:** 10.1111/j.1365-2141.2008.07102.x

**Published:** 2008-05-28

**Authors:** Kazuhiko Natori, Haruka Izumi, Susumu Ishihara, Daisuke N Y Fujimoto, Yasunobu Kuraishi

**Affiliations:** Division of Haematology & Oncology, Department of Medicine, Toho University Medical Centre6-11-1 Oomorinishi, Oota-ku, Tokyo, Japan E-mail: natori@med.toho-u.ac.jp


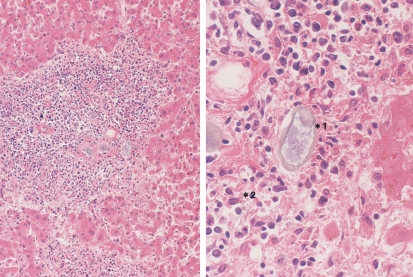


*Schistosoma japonica* invades the human body through the skin with *Oncomelania nosophora*, an intermediate host, and adult schistosoma inhabit and lay eggs in the mesenteric and portal veins. Because of the extinction of *Oncomelania nosophora* in Japan, all new patients with acute schistosomiasis have become infected abroad. We report a case of malignant lymphoma with coexisting *S. japonica* infection. This 72-year-old man, who had experienced general malaise for one month, presented to our hospital with abdominal pain, distention and vomiting. He had cervical lymphadenopathy, conjunctival pallor, scleral icterus and abdominal distention caused by ileus. The liver and spleen were palpable 5 and 4 cm, respectively, below the costal margin. Single 1-cm inguinal lymph nodes were palpable bilaterally. Abnormal test results on admission were: haemoglobin concentration 104 g/l, platelet count 46 × 10^9^/l, total protein 53 g/l, albumin 29 g/l, bilirubin 205 μmol/l, glutamic oxaloacetic transaminase 121 IU/l, lactate dehydrogenase 2460 IU/l. Biopsy of cervical lymph nodes showed non-Hodgkin lymphoma (diffuse large B cell). Abdominal echography showed many hypoechoic areas in the spleen. Liver biopsy showed lymphoma and eggs of *S. japonica* (right and left, *1 *Schistosoma japonicum* egg, *2 infiltrating lymphoma cells). The clinicopathological stage was IVB. The patient did not undergo chemotherapy due to a worsening general condition and abdominal cavity bleeding of unknown origin; he died approximately one month after admission.

Schistosomiasis occurs in Japan, China and the Philippines. *Schistosoma* eggs are most frequently found in the mucosal and submucosal layers of the colon, caecum and sigmoid colon, in that order.

It is hypothesized that the relationship between *S. japonica* and cancer has much in common with that between *Helicobacter pylori* and mucosa-associated lymphoid tissue lymphoma, and that between Epstein–Barr virus and empyema-related lymphoma, in that the mechanisms of chronic inflammation act as cancer-promoting factors. In this case, lymphoma cells proliferated around egg-emboli in Glisson's capsule and portal vein branches, suggesting a close relationship between the infection and the lymphoma.

